# Distinct roles of small extracellular vesicles from resident and infiltrating macrophages on glioma growth and mobility

**DOI:** 10.7150/jca.103595

**Published:** 2025-01-01

**Authors:** Chu-Hsuan Chen, Sheng-Yun Hsu, Wen-Jui Yu, Chi-Shiun Chiang, Ching-Fang Yu

**Affiliations:** 1 Department of Biomedical Engineering and Environmental Sciences, National Tsing Hua University, Hsin-Chu, Taiwan.; 2 Biomedical Technology and Device Research Laboratories, Industrial Technology Research Institute, Hsin-Chu, Taiwan.; 3 Institute of Nuclear Engineering and Science, National Tsing Hua University, Hsinchu, 30013, Taiwan.; 4 The BNCT Research Center, National Tsing Hua University, Hsinchu, 30013, Taiwan.; 5 Department of Medical Imaging and Radiological Sciences, Chang Gung University, Taoyuan, 33302, Taiwan.; 6 Research Center for Radiation Medicine, Chang Gung University, Taoyuan 33302, Taiwan.; 7 Department of Radiation Oncology, Chang Gung Memorial Hospital Linkou Branch, Taoyuan 33382, Taiwan.

**Keywords:** brain tumor, macrophage, microglia, extracellular vesicle, migration

## Abstract

Previous studies revealed that tumor-associated macrophages/microglia (TAMs) promoted glioma invasiveness during tumor progression and after radiotherapy. However, the communication of TAMs with tumor cells remains unclear. This study aimed to examine the role of small extracellular vesicles (sEVs) derived from TAMs in TAMs-mediated brain tumor invasion. This study utilized BV2 and RAW264.7 cell lines representing resident and infiltrating macrophages, respectively, to unveil their effect on tumor cells. Purified sEVs from BV2 and RAW264.7 were validated by nanoparticle track analysis (NTA), transmission electron microscopy (TEM), and western blotting for sEV markers. The effect of sEVs on the murine astrocytoma tumor cell line ALTS1C1 was examined on cell proliferation, migration, and gene expression. The results showed that ALTS1C1 cells effectively engulfed sEVs purified from BV2 and RAW264.7. Only BV2-derived sEVs promoted cell proliferation and were dose-dependent. Further, morphological changes in ALTS1C1 cells were observed after incubation with BV2-derived sEVs, which was associated with enhancing cell migration. BV2-mediated glioma proliferation and mobility were related to the upregulation of vascular endothelial growth factor (VEGF) and downregulation of death effector domain-containing protein (DEDD) gene expression. This study demonstrates the distinct function of sEVs of resident macrophages on glioma cell invasion and reveals the mechanism underlying microglia-mediated tumor progression. These findings suggested resident microglia is the potential therapeutic target for TAMs-induced brain tumor invasiveness.

## Introduction

Glioma is the most common type of tumor originating in the brain. The World Health Organization has categorized glioma into four grades based on integrated classic histological features and molecular biomarkers^1^, ^2^. Glioblastoma, a high-grade astrocytoma, is the most common and malignant glioma. Despite the advances in surgical resection, chemotherapy, and radiotherapy, patients with brain tumors suffer a poor prognosis, and the five-year related survival rate following diagnosis was 35.7%^3^. It is due to high tumor recurrence resulting from the higher invasive ability of brain tumor cells and immunosuppressive microenvironment. Therefore, understanding the interaction of tumor cells with the tumor microenvironment (TME) can promote therapeutic gain and achieve a better tumor control rate.

The TME encompasses the surroundings of a tumor, exerting a significant impact not only on tumor metabolism and growth^4^ but also on the processes of cancer spreading and progression^5^. TME is typically marked by considerable diversity and complexity, consisting of cellular and non-cellular components. Within the TME, various cells are present, including tumor cells, immune cells, fibroblasts, endothelial cells, adipocytes, and neuroendocrine cells, each contributing distinct functions^6^. Non-cellular elements such as the extracellular matrix (ECM), extracellular vesicles (EVs), proteases, and secreted cytokines also interact with tumor cells or immune cells^7^. Additional environmental factors, such as low pH levels^8^, hypoxia^9^, and high interstitial pressure^10^, are also critical determinants of tumor progression and therapeutic outcomes. Over the past decade, the interaction between cells and EVs has gained increasing significance in cancer development and progression^11^.

Recent studies have shown the role of EVs in TME development^12^-^14^. EVs are small bilayered spherical structures generated by almost all mammalian cells. EVs have been widely detected in various biological fluids and show diverse functions and compositions. Through the transfer of bioactive cargo such as proteins, RNAs, and microRNAs, EVs are deeply involved in tumor invasion and metastases^15^. One subtype of EVs, approximately 100-200nm in size, is classified as exosome^16^, which promotes tumor progression and is a critical component of oncogenic transformation^17^. These exosomes are known as one type of “small extracellular vesicles” (sEVs) that are crucial for communication between tumor cells and surrounding cells, such as tumor-associated macrophages (TAMs)^18^.

TAMs are the largest population of infiltrating inflammatory cells in the hypoxic tumor microenvironment^19^, ^20^. They may benefit tumor progression and contribute to chemoresistance^21^, ^22^. TAMs exhibited M1 and M2 phenotypes, which can inhibit or promote tumor growth, respectively^20^, ^23^. In addition, glioblastoma stem cell-derived sEVs can induce M2 macrophage polarization and the expression of programmed death-ligand 1 (PD-L1), which can suppress the immune response^24^. Most previous studies focused on tumor-secreted small EVs and their influence on tumor cells and TME; fewer studies have been conducted on the effects of TAMs-secreted sEVs. While M2 macrophage-derived exosomes are known to interact with tumor cells^25^, the mechanism requires more evidence to declare.

The ALTS1C1 murine astrocytoma cell line, an anaplastic astrocytoma model^21^, has been used to explore changes in the TME during tumor progression and the interaction between tumor cells and TAMs^26^-^28^. The distinct TME within the tumor core and invasion region of ALTS1C1 tumors lead to divergent responses to treatments^26^. TAMs may exhibit different roles during brain tumor progression and radiotherapy^29^, ^30^. Previous evidence also showed that radiation therapy-induced tumor invasiveness was associated with macrophage mobilization and vasculogenesis^28^. Our recent study also demonstrated that microglia protected brain tumor cells from the toxicity of therapeutic drugs through gap junction. Blocking gap junction reduced the microglia-mediated chemoresistance^31^. Therefore, understanding the interaction between tumor cells and TAMs can enhance therapeutic efficacy and achieve better outcomes. Recent studies highlight the key role of EVs in the communication between tumor cells and stroma cells. While tumor cells-derived sEVs are well studied, the roles of sEVs from macrophages or microglia in TME remain relatively unexplored. This study focused on the role of macrophage/microglia-derived EVs in brain tumor progression and aimed to examine the interaction between macrophage/microglia and tumor cells *in vitro*. This study explored the impact of sEVs derived from infiltrating macrophages (RAW264.7) and resident macrophage (BV2) on ATLS1C1 cells, focusing on the changes in cell morphology, proliferation, and mobility while elucidating potential underlying mechanisms.

## Material and Methods

### Cell culture

The murine astrocytoma cell line ALTS1C1 (BCRC, Taiwan)^21^, macrophage cell line RAW264.7 (ATCC, USA), and murine microglia cell line BV2 (ICLC, Genova) were maintained in Dulbecco's modified Eagle's medium (Gibco, NY, USA) supplemented with 10% fetal bovine serum (Gibco) and 1% penicillin-streptomycin (Gibco) at 37°C and 5% CO_2_ in a humidified atmosphere. Mycoplasma contamination was assessed using the EZ-PCR™ Mycoplasma Detection Kit (Biological Industries, Israel) before experiments.

### Orthotopic brain tumor implantation

ALTS1C1 cells (1×10^5^) were implanted intracranially into 8-10 week C57BL/6J mice (IACUC No.:107042). The intracranial tumor implantation procedure followed the protocol detailed in a previous study^21^. Mice were euthanized upon displaying signs of neurological impairment, such as lethargy, inability to ambulate, or a body weight loss exceeding 20%. Brain tumor samples were then extracted, embedded in OCT compound (Sakura Finetek), and stored at -80°C.

### Immunohistochemistry staining

Frozen tissue sections were fixed in cold methanol and stained with antibodies targeting CD68 (Abcam) and TMEM119 (Abcam). Primary antibody binding was visualized using secondary antibodies conjugated with Alexa Fluor 594 or Cy5 (Invitrogen).

### Small EVs isolation

Cells were grown to 70% confluency, washed with PBS to remove FBS, and then cultured in DMEM with 10% exosome-depleted FBS (Gibco) for 24 hours. Cell debris and apoptotic bodies were removed by gradient centrifugation. The supernatant was filtered using a 0.22 μm PVDF filter (Merck, Darmstadt, Germany) and concentrated with an Amicon® Ultra-15 3K filter tube (Merck). Small EVs were purified using ExoQuick-TC (System Biosciences, CA, USA) and incubated at 4°C for 12 hours. After centrifugation (1500×g, 30 minutes), the pellets were resuspended in PBS and stored at -80°C^32^.

### Nanoparticle Tracking Analyzer (NTA)

The sEV solution was diluted 100 to 200-fold in particle-free PBS, following the manufacturer's guidelines. The nanoparticle size distribution was analyzed using NanoSight NS300 under constant flow conditions (flow rate=50) at 25°C. Three videos (60-second length) were captured at 25 frames per second with a camera level of 14. Data analysis was performed using NTA3.2 software, which was optimized to identify and track each particle frame by frame. The concentration (particles/ml) of the purified sEVs was also determined by NTA simultaneously. Every batch of purified sEVs was confirmed by NTA to control the quality of sEVs for the following experiments.

### Transmission Electron Microscopy (TEM)

sEVs (1×10^9^ particles) were fixed in 200 μl of 2.5% Glutaraldehyde (Sigma, MA, USA) at 4°C for 15 minutes. The fixable sEV solution was loaded onto TEM grids (Ted Pella, CA, USA). After a 30-minute incubation, the excess solution was removed, and the grids were stained with 1% phosphotungstic acid hydrate (Sigma) for 2 seconds. The TEM grids were dried at 65°C for three days, and the images were captured by a TEM microscope (HITACHI, Tokyo, Japan).

### Western blotting

The sEV pellets were lysed by T-PER (Thermo, MA, USA) with protease inhibitor (Roche) and the concentration was determined by BCA assay (Sigma). Protein (10 μg) was mixed with 4×loading buffer, boiled for 5 minutes, and run on a 15% SDS-PAGE gel at 150V. Proteins were transferred to a PVDF membrane, blocked with Immobilon® Block (Merck) for 1 hour, and incubated overnight at 4°C with primary antibodies (CD63, TSG101, CD9; Abcam). After 1 hour with HRP-conjugated secondary antibodies, the membrane was treated with chemiluminescent HRP substrate (Merck), and images were captured using a Syngene G Chemi XT4 (Syngene International Limited, India).

### Uptake of Fast Dio-labeled sEV

RAW264.7 and BV2 cell-derived sEVs were labeled with Fast Dio™ (Thermo) at 37°C for 30 minutes. After removing excess dye by ExoQuick-TC, Dio-labeled sEVs were incubated with ALTS1C1 cells for 24 hours, and images were captured using a ZEISS microscope (Germany) and flow cytometry.

### Cell proliferation assay

ALTS1C1 cells (2000 cells) were incubated with sEVs with serial dilution from 1×10^10^ particles/ml to 1×10^7^ particles/ml in a 96-well plate. After three days of incubation, cell proliferation was measured by MTT assay (Sigma) at an absorbance of 570 nm.

### Cell cycle analysis

ALTS1C1 (1×10^5^ cells) were co-incubated with sEVs (2.5×10^9^ particles/ml) in 12 well culture plates. The cell numbers were calculated 24, 48, and 72 hours after incubation. Cells were fixed in cold 70% ethanol solution for 30 minutes. Then, the PI solution (Sigma) and RNase A (Thermo) were added and incubated at 37°C for 15 minutes. Cell cycle analysis was performed on BD FACS Canto (BD Bioscience), and the data were analyzed by Cell cycle analyzer in FlowJo software version 10 (BD Bioscience).

### Wound healing assay

ALTS1C1 (1×10^5^ cells) were seeded in the wound healing assay inserts (Abcam) containing sEVs (2.5x10^9^ particles/ml). After 24 hours, the insert was removed, and the cells were incubated for another 16 hours. The images were captured by the microscope, and the width of the wound was measured by Fiji ImageJ software.

### Migration assay

ALTS1C1 cells (1×10⁵) were incubated with sEVs (2.5×10⁹ particles/ml) for 24 hours and then seeded in 8-μm Transwell chambers (Corning, USA) to assess mobility. Cells (1×10^5^ cells) were added to the upper chamber in 2.5% FBS medium, while the lower chamber contained complete medium. After 16 hours, non-invading cells were removed, and the invading cells were counted under a microscope.

### RT-PCR

ALTS1C1 (1×10^5^ cells) were co-cultured with sEVs (2.5x10^9^ particles/ml) for 24 hours. Total RNA was extracted using the RNeasy® Mini Kit (Qiagen, Hilden, Germany) and quantified by Nanodrop 2000 (Thermo). Target gene expression was detected by KAPA SYBR® FAST One-Step qRT-PCR Master Mix (2×) Kit (Merck) and analyzed by the StepOne™ Real-Time PCR System (Thermo). GAPDH was used as the housekeeping gene, and the fold change of target genes in each group was calculated as 2^(-ΔΔCT) compared to control. The primer sequences of each gene are described in Table 1.

### Statistics

All experiments were performed in triplicate, and data were analyzed using GraphPad Prism. Statistical significance was determined with p-values < 0.05 using unpaired t-tests or one-way ANOVA. *p<0.05, **p<0.01, ***p<0.001,****p<0.0001.

## Results

### TMEM119+ microglia were aggregated in invasive islands

**Figure 1:**
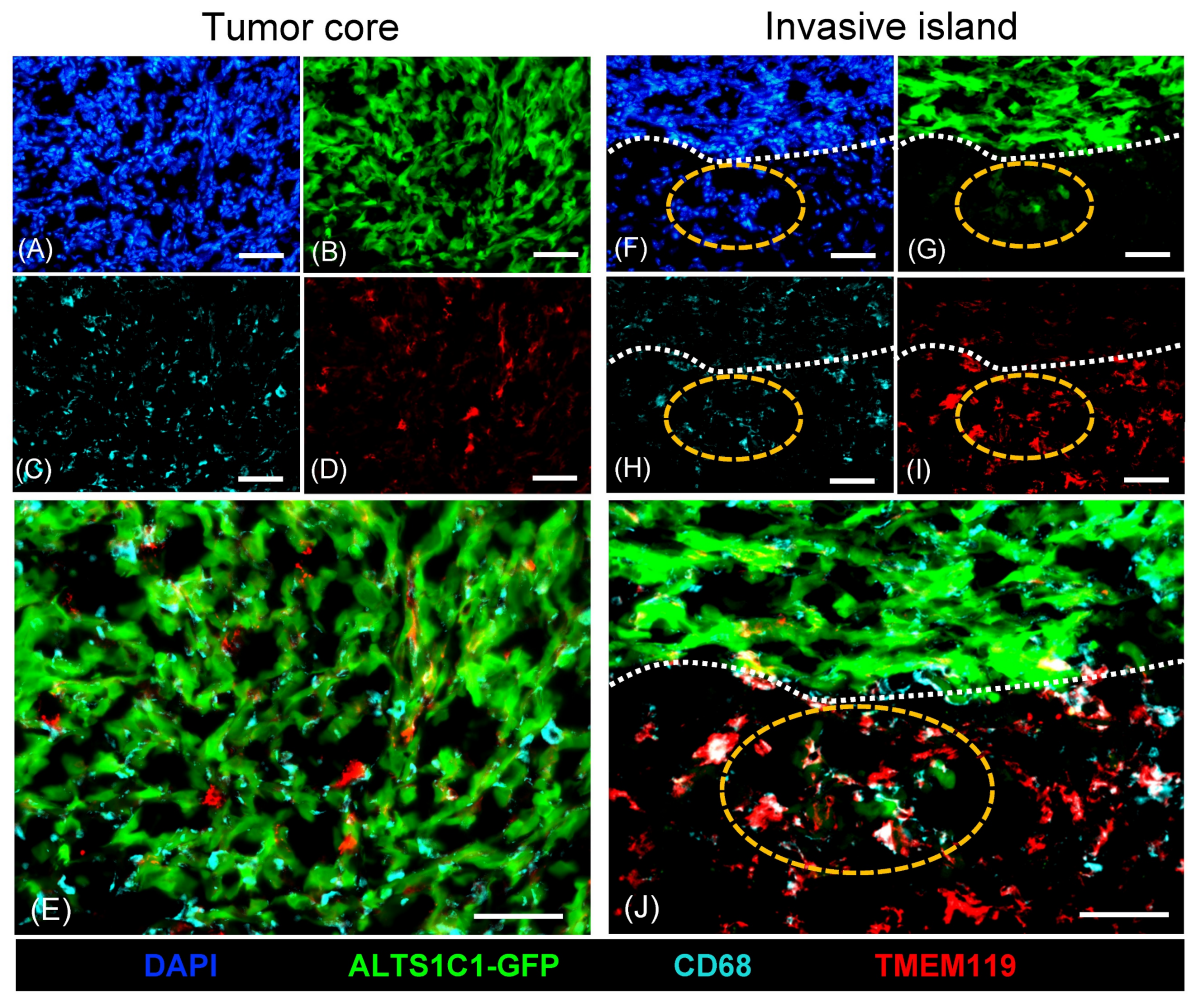
** Different distribution of CD68+ macrophages and TMEM119+ microglia in ALTS1C1 tumors.** ALTS1C1-GFP tumors (B, G) were stained with antibodies of macrophage CD68 (C, E, H, J) and microglia TMEM119 (D, E, I, J). Nuclei were visualized by DAPI (A, F). The invasive island and tumor edge were marked by orange and white dot lines, respectively. Scale bar= 50μm.

A previous study has unveiled the presence of abundant microglia surrounding tumors during ALTS1C1 brain tumor progression^31^. This study further explored the role of macrophages/microglia within brain TME. Tumors were simultaneously analyzed by staining with general macrophage marker CD68 and microglia-specific marker TMEM119 (Figure 1). Immunohistochemistry staining showed a significant presence of CD68+ macrophages infiltrating the tumor core (Figure 1C, E), while only a few TMEM119+ microglia were found (Figure 1D, E). Consistent with the previous study, more TMEM119+ microglia cells were presented in tumor surrounding tissues (Figure 1I). Notably, TMEM119+ microglia aggregated within invasive islands (Figure 1I) and were closely associated with GFP-expressing tumor cells (Figure 1J). These findings indicated the heterogeneous distribution of subtypes of macrophages within brain TME. CD68+ infiltrating macrophages were predominantly recruited to the tumor core, whereas TMEM119+ resident macrophages (microglia) were mainly clustered in invasive islands. This evidence leads to a hypothesis that microglia are pivotal in promoting tumor invasiveness during tumor progression.

### Characterization of purified extracellular vesicles

**Figure 2:**
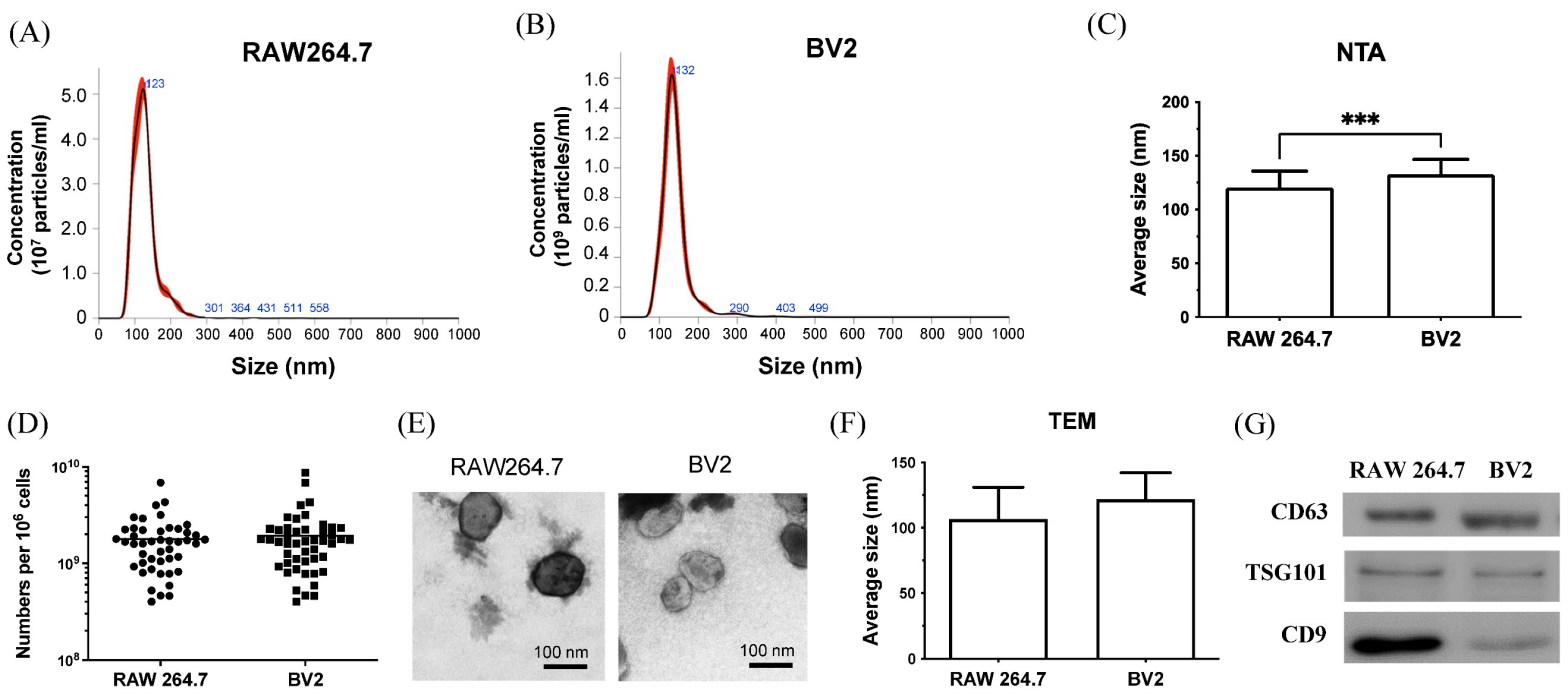
** Characterization of sEVs purified from RAW264.7 and BV2 cells.** (A-B) The size distribution of the sEVs purified from RAW264.7 and BV2 cells was measured by NTA analysis. (C) The average size and (D) the total amount of sEVs were quantified. (E) The representative TEM image of sEVs. Scale bar: 100 nm. (F) The size of sEVs measured by TEM images. N= 6 images/group. (G) The protein component of sEVs analyzed by western blot.

To prove this hypothesis, this study adapted a previously established *in vitro* co-culture model^31^ to examine further the interaction of tumor cells and macrophage/microglia in tumor growth and mobility. Recent reports highlight the importance of EVs in the communication between tumor cells and stroma cells. However, the roles of EVs from macrophages or microglia in TME remain unclear. To assess the function of EVs from infiltrating macrophages and resident microglia on brain tumor progression, EVs from RAW264.7 and BV2 cells are purified and analyzed for size, concentration, and protein using NTA, TEM, and western blot. NTA was used to analyze the size distribution and concentration of purified EVs^33^. The results show that the size of the purified EVs is approximately 120-130 nm (Figure 2A-B). The average size of BV2-derived EVs was larger than that of RAW264.7-derived EVs (Figure 2C), with both cells releasing comparable amounts of EVs (Figure 2D). TEM result confirmed the size and round shape of EVs (Figure 2E), with average sizes of 106.7 ± 24.22 for RAW264.7 and 121.7 ± 20.41nm for BV2, which was consistent with NTA data (Figure 2F). Based on the results of NTA and TEM images, the purified EVs were classified as “small EVs (sEVs)”, a subpopulation of EVs^34^. Western blot analysis (Figure 2G) showed that sEVs from RAW264.7 and BV2 contained the general protein marker CD63 and the sEVs-specific marker TSG101. The cell type-specific protein CD9, expressed by mesenchymal stem cells, was also detected in both sEVs. In conclusion, NTA, TEM, and western blot results confirmed that the isolated vesicles were sEVs and expressed sEVs-related markers.

### Purified sEVs had high efficiency in being engulfed by ALTS1C1 cells

**Figure 3:**
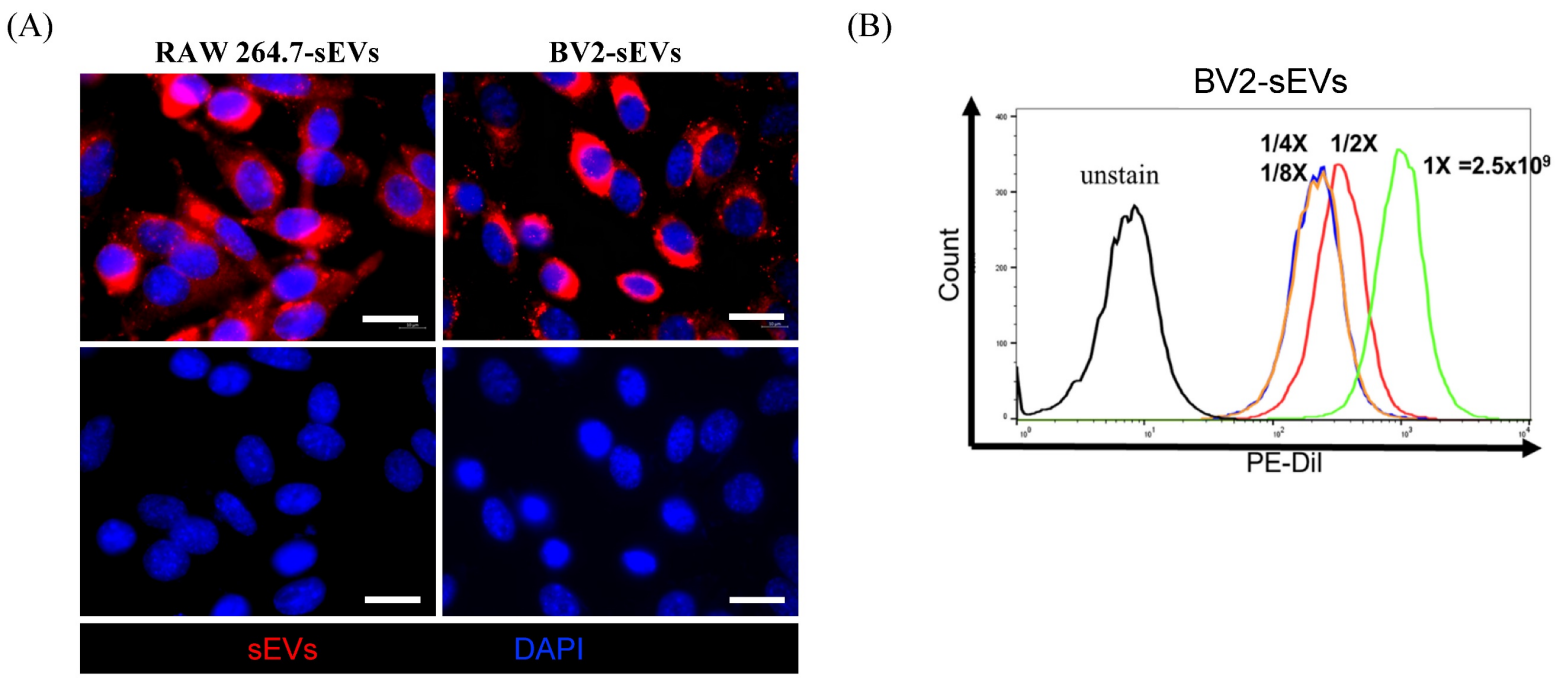
** Cellular uptake of BV2 and RAW264.7-derived sEVs.** Purified sEVs labeled with Dil dye were uptake by ALTS1C1 cells. The immunofluorescence images for ALTS1C1 cells co-incubated with BV2/RAW264.7-derived sEVs (red) for 24 hours. The nuclei were visualized by DAPI. Scale bar: 20 μm. (B) The intensity of dye was analyzed by flow cytometry after co-incubation with different particles of BV2-sEVs.

To assess the uptake of sEVs by recipient cells, donor cell-derived sEVs were labeled with FAST Dil dye and co-cultured with the ALTS1C1 recipient cells. The sEVs uptake by ALTS1C1 cells was confirmed under fluorescence microscopy. Figure 3A shows the results of ALTS1C1 cells incubated with Dil-labeled sEVs derived from RAW264.7 and BV2 cells for 24 hours. The Dil-labeled sEVs were localized in the cytoplasm of ALTS1C1 cells, indicating that sEVs derived from RAW264.7 and BV2 cells can be engulfed by ALTS1C1 cells. To further assess the internalization of sEVs, ALTS1C1 cells were incubated with BV2-derived sEVs labeling with Dil dye for 24 hours, and the ability of ALTS1C1 to uptake sEVs with different particle numbers was confirmed by flow cytometry. The results showed that a high-intensity signal of Dil dye was detected after incubation with sEVs, indicating that ALTS1C1 cells had a good ability for sEVs internalization, and the uptake of sEVs by ALTS1C1 cells was dose-dependent (Figure 3B). Therefore, purified sEVs from RAW264.7 and BV2 cells were used in the following assays to evaluate the effect on ALTS1C1 tumor cells.

### BV2-derived sEVs had a dose-response effect on cell proliferation of brain tumors

**Figure 4:**
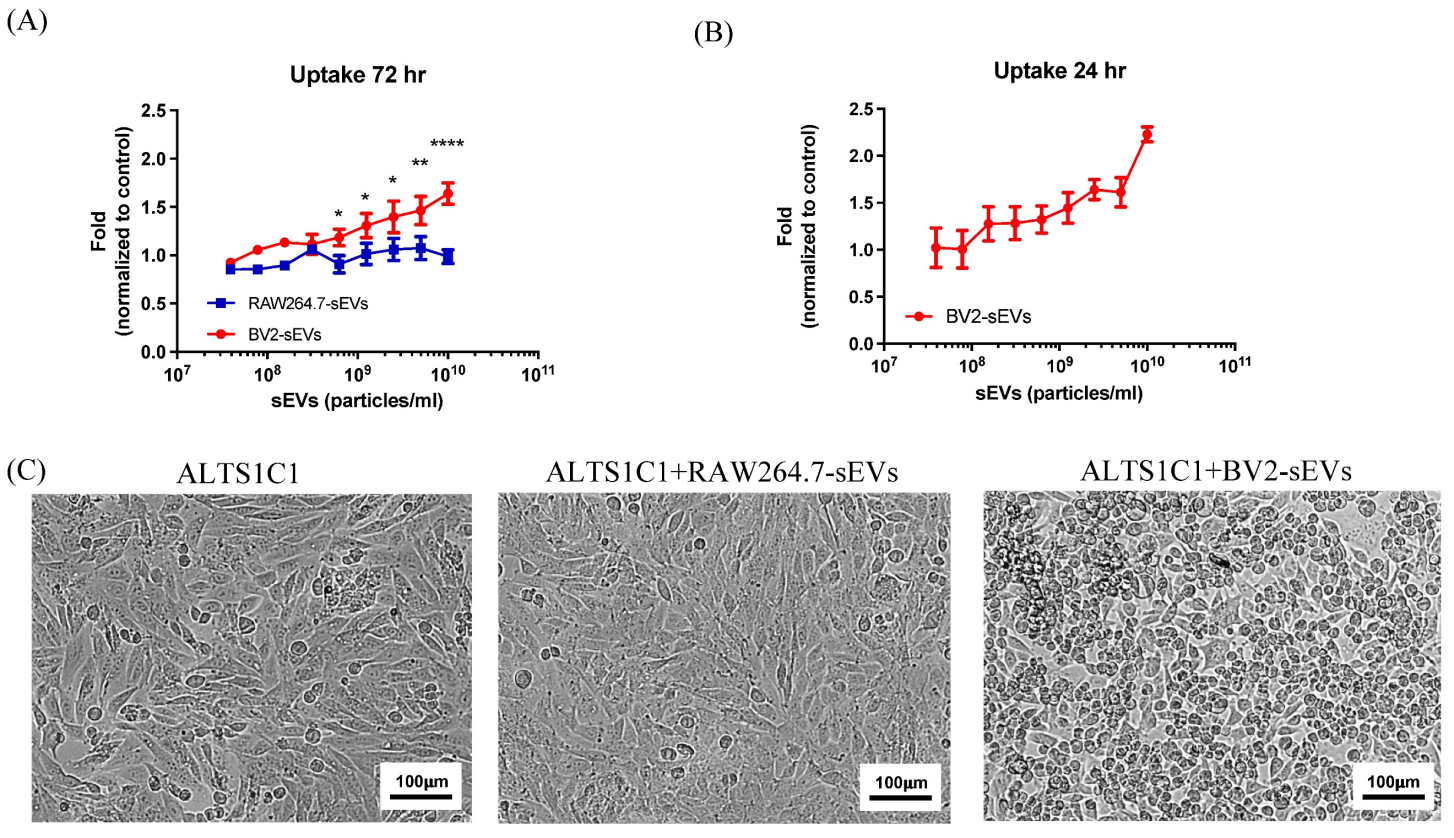
** BV2-derived sEVs mediated long-term effect on ALTS1C1 cell proliferation.** (A-B) Fold change of sEVs-mediated proliferation was assayed for 72 hr- and 24 hr-incubation, respectively, and cell proliferation was measured by MTT assay at 72 hours. N= 3 in each dose. (C) The representative images of ALTS1C1 with sEVs-trestment for 24 hrs. Scale bar: 100 μm.

To investigate the effects of BV2-derived sEVs (BV2-sEVs) and RAW264.7-derived sEVs (RAW264.7-sEVs) on cell proliferation, the growth of ALTS1C1 cells was examined by treatment with various numbers of sEVs for 3 days. The result reveals that the cell number of ALTS1C1 cells had a significant increase after incubation with BV2-sEVs, especially in higher concentrations, and was dose-dependent (Figure 4A). On the other hand, no significant change in ALTS1C1 cell growth was observed in the incubation with RAW264.7-sEVs group. To further verify that the long-term stimulation of BV2-sEVs is essential for promoting cell proliferation, ALTS1C1 cells were co-cultured with different numbers of BV2-sEVs for 24 hours. Then, the cell medium was replaced by an sEV-free cell culture medium for another 48 hours of incubation. As shown in Figure 4B, an increase in cell growth of ALTS1C1 was also observed and was dose-dependent. Treatment of sEVs also affected the morphology of ALTS1C1 cells. Under standard culture conditions, ALTS1C1 cells exhibit a spindle-like morphology. Following treatment with BV2-sEVs, more ALTS1C1 cells displayed a rounded morphology, whereas treatment with RAW264.7-sEVs did not induce such changes (Figure 4C). These results indicated that BV2-sEVs, compared to RAW264.7-sEVs, uniquely stimulated ALTS1C1 cell proliferation and morphological changes.

### BV2-derived sEVs caused the morphological change of ALTS1C1 cells

**Figure 5:**
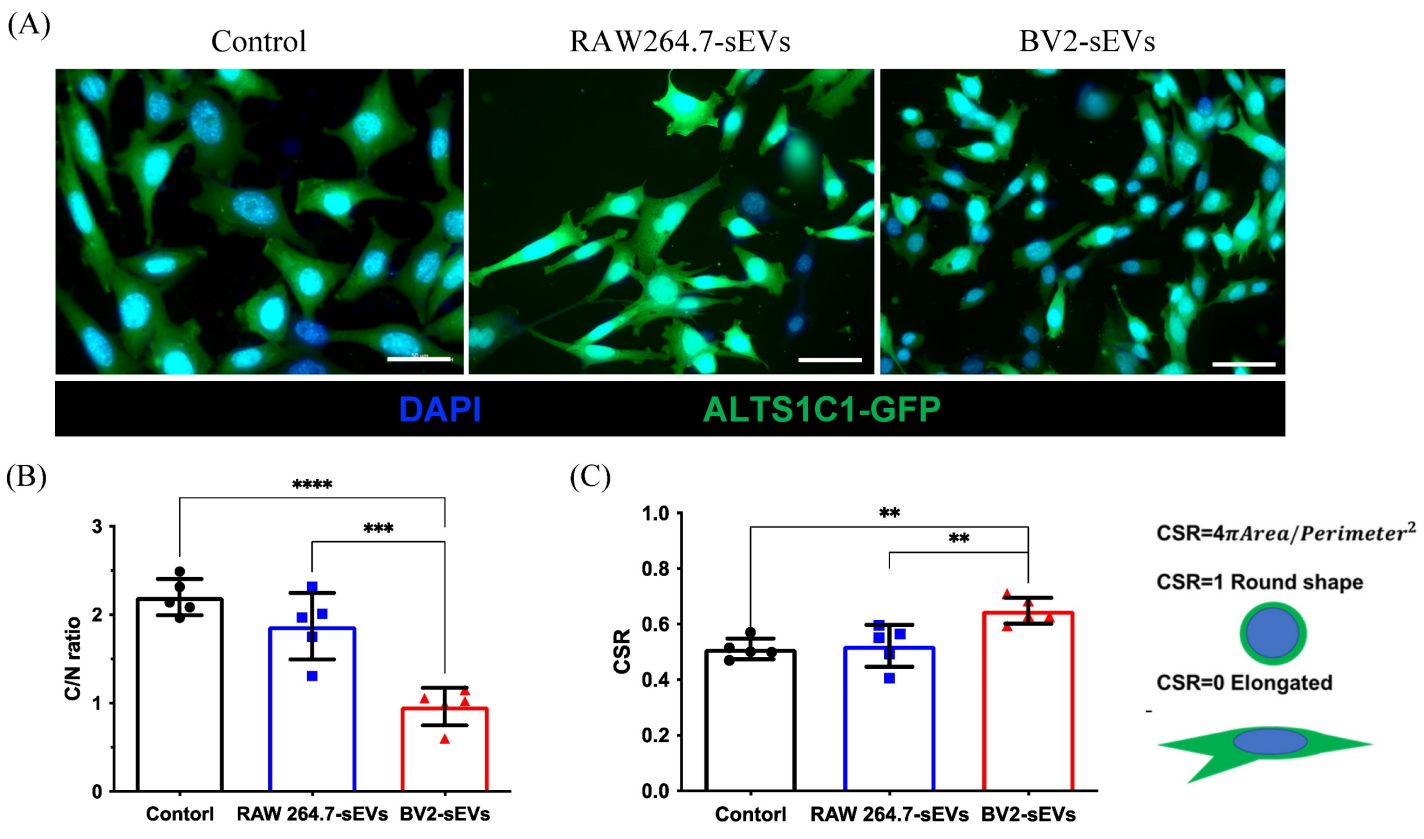
** BV2- derived sEVs caused ALTS1C1 cell morphological change.** (A) The immunofluorescence images for ALTS1C1-GFP cells incubated with sEVs for 24 hours. Scale bar: 50μm. The images were quantified by (B) C/N ratio and (C) CSR. N=5 field per group.

To further verify the morphological change of ALTS1C1 cells caused by BV2-derived sEVs (Figure 4C), the cell size and shape were quantified after sEVs treatment. The green fluorescence protein-expressed ALTS1C1 cells (ALTS1C1-GFP) was used to identify the morphology of cells. The results showed that the size of ALTS1C1 cells after BV2-sEV treatment was smaller than the control or RAW264.7-sEV-treated group (Figure 5A). The size of the ALTS1C1 cells was assessed by the cytoplasmic area ratio to the nucleus area (C/N ratio). The C/N ratio of the control group is 2.198 ± 0.206, indicating a large cytoplasmic area existed in standard culture conditions. RAW264.7-sEVs treatment slightly reduced the C/N ratio (1.870 ± 0.375) but had no significant difference from the control group. However, the cytoplasmic area in the BV2-sEVs group (0.960 ± 0.212) was significantly reduced compared to the control and RAW264.7-sEVs groups, indicating that BV2-sEV treatment caused cell contraction and became smaller than those in the other two groups (Figure 5B). The morphological change of ALTS1C1 cells after sEVs treatment was further assessed by the cell shape ratio (CSR). The CSR ratio was an indicator to determine the shape of the cells and was defined as the ratio of 4πArea to (perimeter)^2^. The ratio of CSR is approximately 1 if the cell is round. Otherwise, the CSR is approximately 0, which presents an elongated and spindle-like morphology. The quantified results showed that, in standard conditions, the ratio of CSR of ALTS1C1 cells is 0.511 ± 0.037. After 24 hours of incubation, RAW264.7-sEVs did not show significant alteration in the shape of ALTS1C1 cells (0.522 ± 0.075) compared to the control group. BV2-sEVs remarkably increased the ratio of CSR of ALTS1C1-GFP cells (0.648 ± 0.047), indicating a tendency for ALTS1C1 cells to adopt a rounded morphology. These results demonstrated that BV2-derived sEVs can induce the morphological change of ALTS1C1 cells (Figure 5C).

### BV2-derived sEVs promoted cell proliferation and migration ability of ALTS1C1

**Figure 6:**
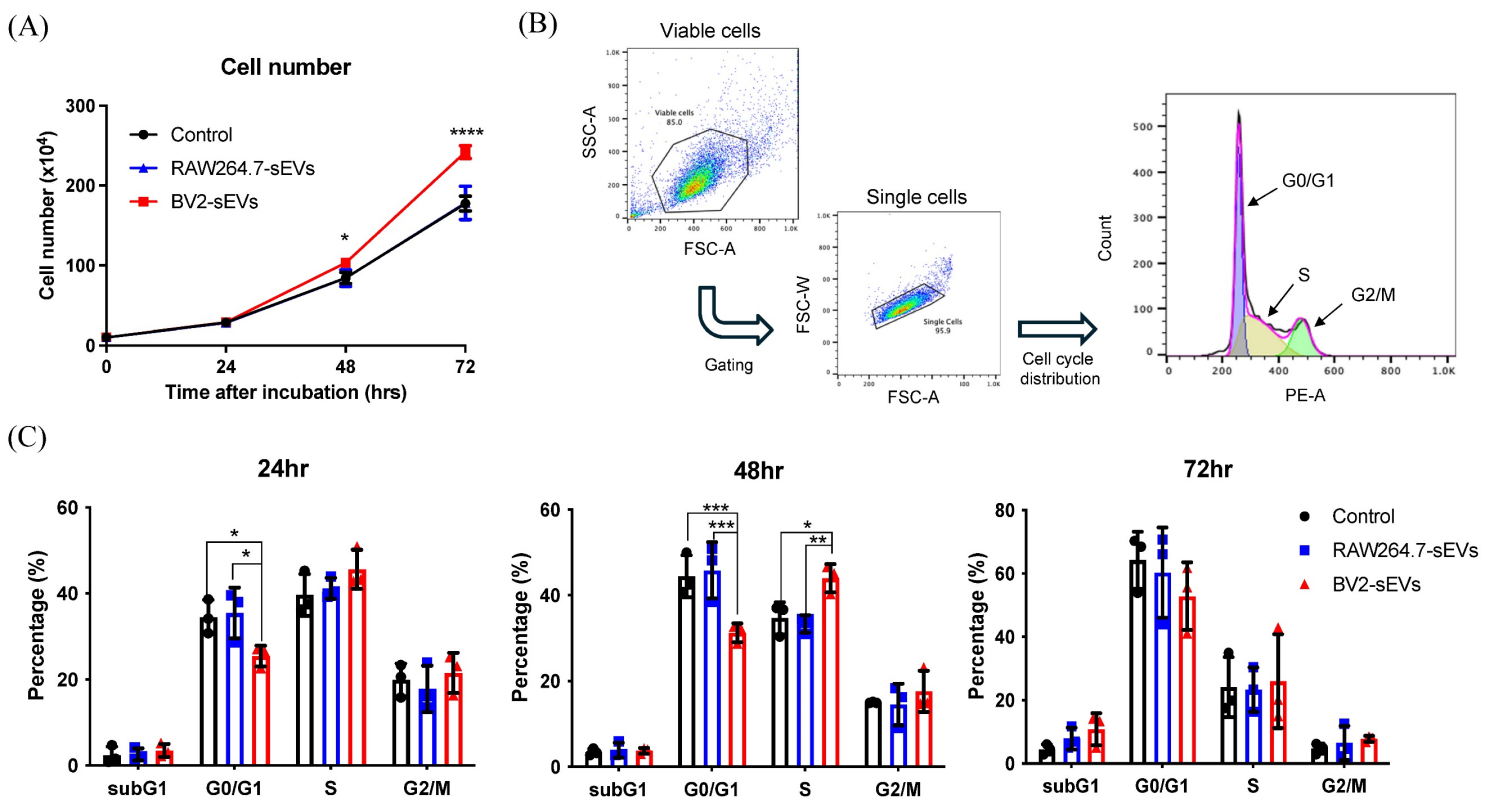
** BV2-derived sEVs induced ALTS1C1 cell proliferation.** ALTS1C1 cells were incubated with RAW264.7/BV2-derived sEVs at different times. (A) The growth curve of the ALTS1C1 cells incubated with sEVs. (B)The gating strategy of cell cycle analysis. (C) The percentage of cell phase in 24, 48, and 72 hours after cultured with sEVs. N=3 per group.

The above data indicated that BV2-sEVs could prompt a change in ALTS1C1 cell shape, leading to an approximate rounded morphology. The rounding up of adhesion cells is usually associated with the tendency of cell migration or entering the M phase of the cell cycle^35^, ^36^. To further investigate the impact of BV2-derived sEVs on cell proliferation of ALTS1C1 cells, cell numbers of sEV-treated ALTS1C1 were examined after treatment. The result showed a significant increase in the cell number when treated with BV2-sEVs after 48 and 72 hours (Figure 6A). No difference was observed between RAW264.7-sEVs and control group. The percentage of the cell population was further examined by the cell cycle analysis at 24, 48, and 72 hours (Figure 6B). Following a 24-hour incubation period, the percentage of cells in the G1 phase decreased after treatment with BV2-sEVs. Moreover, the reduction in the G1 phase was notably more pronounced after 48 hours of incubation compared to the control and RAW264.7-sEVs group. The percentage of S phase in BV2-sEV treated ALTS1C1 cells increased significantly compared to those of the control or RAW264.7-sEV-treated group at 48 hours of incubation (Figure 6C). There was no difference between each group at 72 hours of treatment. These results indicated that BV2-derived sEVs could stimulate ALTS1C1 cell proliferation.

**Figure 7:**
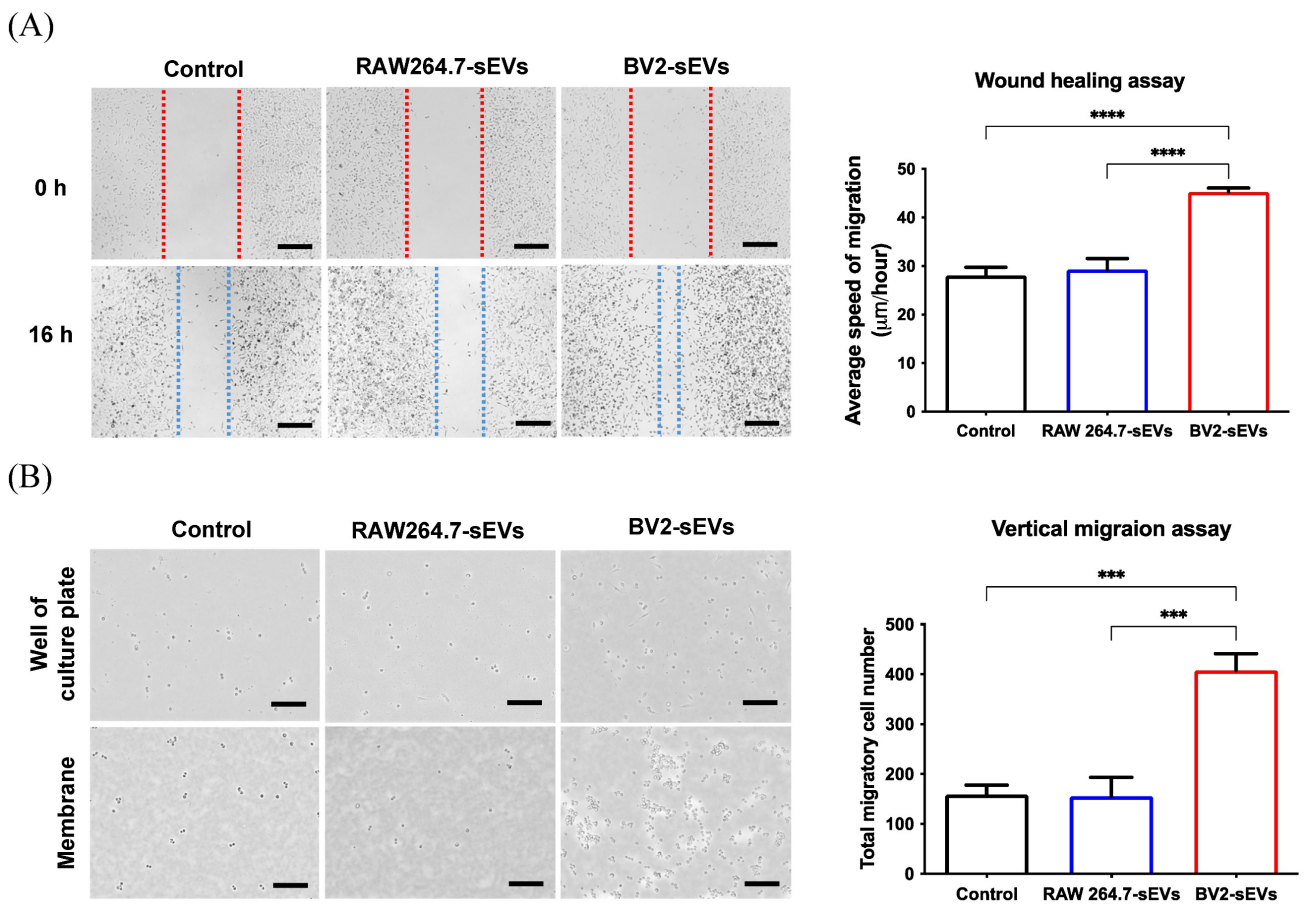
** BV2-derived sEVs enhanced cell mobility of ALTS1C1 cells.** ALTS1C1 cells were incubated with sEVs for 24 hours, and cell mobility was examined by wound healing assay and migration assay. (A-B) The representative images and the quantification of wound healing assay and migration assay. Scale bar: 500μm (A) and 200μm (B). N=3-4 per group.

The migration ability of ALTS1C1 cells was further evaluated using wound healing and migration assay. The results of wound healing assay showed that the migration rate of the BV2-sEV-treated group was remarkably higher than those of the control and the RAW264.7-sEV-treated groups (Figure 7A). The vertical migration assay showed that more ALTS1C1 cells were facilitated to pass through 8-μm pores and migrate to the bottom plate after 16 hours (Figure 7B). The migrated cell numbers of the BV2-derived sEV-treated group increased significantly than the control and RAW264.7-sEVs groups. These results indicated that BV2-derived sEVs could enhance the migration ability of ALTS1C1 cells.

### BV2-derived sEVs altered the expression levels of VEGFa and DEDD in ALTS1C1 cells

**Figure 8:**
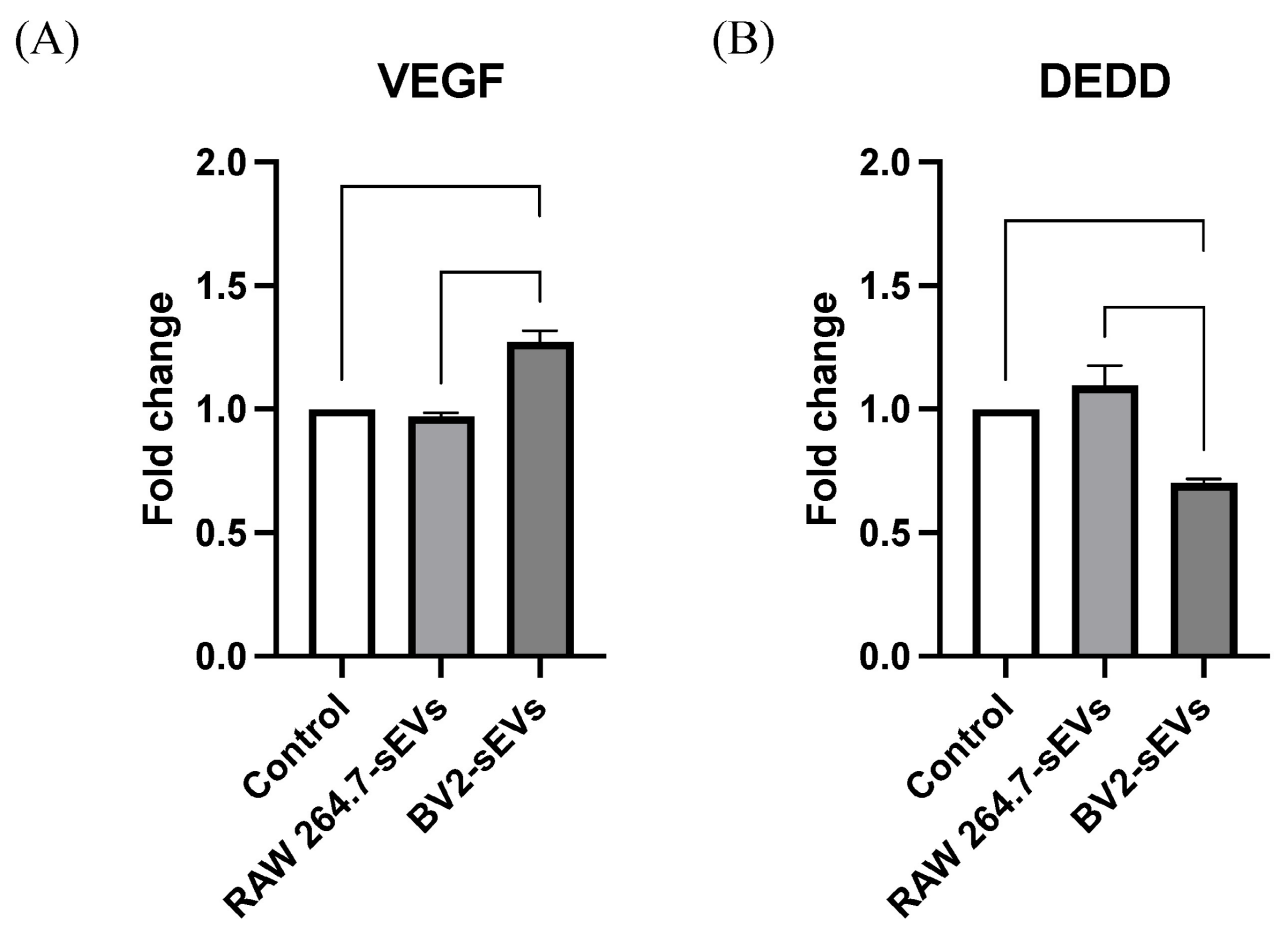
** VEGF and DEDD expression of ALTS1C1 cells were altered by BV2-derived sEVs.** The levels of *VEGFa* and *DEDD* in ALTS1C1 cells were quantified after incubation with sEVs for 24 hours. N= 3-5 per group.

To investigate the mechanism underlying the cell proliferation and mobility of ALTS1C1 cells affected by BV2-derived sEVs treatment, the expression of vascular endothelial growth factor-A (VEGF-A) and death effector domain-containing protein (DEDD), which are related to cell cycle and migration, in EVs-treated ALTS1C1 cells were analyzed by RT-PCR. The results indicated that treatment with BV2-sEVs significantly upregulated VEGF-A levels by approximately 1.27-fold in ALTS1C1 cells and decreased the expression of DEDD by approximately 0.7-fold (Figure 8). This evidence suggested that BV2-derived sEVs might regulate cell proliferation and migration of ALTS1C1 cells through the signal transduction pathways of VEGF-A and DEDD genes.

## Discussion

Tumor-associated macrophages support tumor progression by promoting immunosuppression, invasion, metastasis, and angiogenesis. Previous studies indicated that inhibition of stromal cell-derived factor 1 (SDF-1) expression reduces macrophage recruitment in ALTS1C1 tumor cells, thereby highlighting the crucial role of macrophages in brain tumor growth^21^. The present study showed the heterogeneous spatial distribution of subtypes of macrophages in brain TME. CD68+ infiltrating macrophages were predominantly in the tumor core, and TMEM119+ resident macrophages (microglia) were clustered in invasive islands. Further, this study revealed the distinct role of the sEVs from the infiltrating macrophages and resident microglia on brain tumor growth and mobility by an *in vitro* model.

Recent studies reported that EVs are highly associated with tumor growth, invasion, metastasis, and treatment resistance^37^. To elucidate the involvement of sEVs in macrophage-brain tumor cell interaction, the effect of sEVs derived from macrophages RAW264.7 and microglial BV2 on the growth and migratory capability of ALTS1C1 cells was investigated *in vitro*. This study demonstrated that sEVs derived from BV2 cells, but not RAW264.7 cells, had a crucial influence on ALTS1C1 cells. BV2-derived sEVs facilitated ALTS1C1 cell proliferation and promoted cell mobility. In addition, BV2-derived sEVs upregulated VEGF-A expression and reduced DEDD expression of ALTS1C1 cells, which might participate in the regulation of cell growth and mobility. This study demonstrated that brain resident microglia significantly impacted tumor growth and migration via releasing sEVs and provided direction for designing new strategies to enhance therapeutic efficiency for brain tumors.

Extracellular vesicles serve as carriers for molecular communication between cells. Glioblastoma cells release exosomes to promote cell viability, invasion, and radioresistance of recipient cells^38^, and, after irradiation, glioblastoma cells enhance cell mobility by releasing connective tissue growth factor (CTGF) mRNA-abundant exosomes^39^. Tumor cells-derived EVs can also affect tumor stroma cells to modulate the tumor microenvironment. Glioblastoma cell-derived EVs upregulated the expression of the pro-tumor genes in microglia cells, including the CXCL1/10, CCL2/CCL5, and IL-6, and downregulated immune response-associated genes, such as IL-16, IL-23, and IL-27^40^. Under hypoxia, glioma-derived exosomes had a higher ability to stimulate the expansion and activation of myeloid-derived suppressor cells via miR-10a/Rora and miR-21/Pten pathways^41^. The elevated levels of EVs in glioblastoma patients' plasma imply a crucial role in advancing brain tumors^42^. In recent years, TAM-derived exosomes have received growing attention in cancer therapy. TAM-derived exosomes promoted tumor proliferation, invasion and metastasis, angiogenesis, immune response, drug resistance, and tumor metabolism^43^. Yin *et al.* demonstrated that TAM-derived exosomes promote pancreatic ductal adenocarcinoma (PDAC) cell invasion and migration by miR-501-3p-mediated TGFBR3/TGF-β signaling pathway^44^. High levels of miR-95 in TAMs-derived exosomes promoted prostate cancer progression by inducing the proliferation and epithelial-mesenchymal transformation via binding to its target gene *JunB*^45^. Chuang *et al.* demonstrated that glioma-associated macrophages (GAM)-derived exosomes increased the resistance to temozolomide (TMZ) by modulating STAT3/miR-21/PDCD4 pathway. Inhibiting the STAT3 pathway reduced exosome release and the miR-21-5p level of GAM-derived exosomes, indicating its potential as an adjuvant to TMZ therapy^46^. The present study used RAW264.7 and BV2 cell lines to demonstrate that BV2-derived sEVs could improve cell proliferation and enhance the migratory ability of brain tumor cells. These findings indicated that sEVs are essential in the interaction between brain tumor cells and macrophages/microglia. Targeting sEVs-associated molecules and their downstream pathways pave a new avenue for controlling brain tumor progression.

This study shows that BV2-derived sEVs altered the level of VEGF-A and DEDD in ALTS1C1 tumor cells, which might be associated with the BV2-mediated enhancement of migration and proliferation of ALTS1C1. VEGF-A can promote the proliferation and migration of endothelial cells^47^ and induce the migration of cancer cells^48^. DEDD is known to promote tumor cell apoptosis and inhibit tumor proliferation^49^ and is also the target of miR-24-3p, which regulates bladder tumor proliferation and migration^50^. A previous study demonstrated that extracellular vesicles derived from BV2 cells contained a high level of miR-24-3p^51^, which might be the critical factor of BV2-derived sEVs modified the expression of DEDD and VEGF-A in ALTS1C1 cells at the post-transcriptional state. miR-24-3p has been found to reduce DEDD gene expression and subsequent protein levels, thus promoting tumor proliferation and migration^50^. miR-24-3p also induces VEGF-A expression in human astrocytoma^52^. The present study showed sEVs from BV2 cells upregulated VEGF-A and downregulated DEDD expression of ALTS1C1 brain tumor cells. These findings are consistent with the results of previous studies, suggesting the microglia cells promoted brain tumor progression via EVs communication, which might be mediated through miR-24-3p-associated pathways.

Microglia, the resident immune cells in the central nervous system (CNS), are essential to brain homeostasis and pathogen defense but are also implicated in CNS disease and tumors. However, the similarity in cellular markers makes it difficult to distinguish infiltrating macrophages from resident microglia, thereby obscuring the role of microglia in brain cancer growth and treatment. Recently, TMEM119 was identified as a unique marker expressed only in microglia^53^. Jiang *et al.* demonstrated that CD163 and TMEM119 were positively correlated with microvessel density. The higher CD31 expression had a shorter median survival time in glioma patients^54^. These results suggested M2 microglia were positively correlated with the density of microvessels in GBM patients, leading to a lower survival rate. The previous study also showed that many TMEM119+ microglia cells were recruited and surrounded the brain tumors in a murine astrocytoma model. The* in vitro* assay demonstrated microglia promoted tumor chemoresistance via gap junction connection, indicating microglia had participated in tumor growth and resistance to treatment^31^. Therefore, understanding the roles of microglia will provide critical strategies for treating gliomas. To date, numerous studies have demonstrated the crucial impact of microglial exosomes on brain cancers. M2-polarized HMC3 microglia promoted tumor angiogenesis by releasing exosomal circKIF18A, and circKIF18A bound to its target gene FOXC2 that upregulated ITGB3, CXCR4, and DLL4 expressions and activated PI3K/AKT signaling^54^. Another study reported that M2-polarized BV2 microglial released exosomes for transferring miR-7239-3p to glioma cells that downregulated Baml1 tumor suppressor gene expression, promoting glioma proliferation and migration^55^. An interesting study showed that exosomes from Toxoplasma gondii-infected microglia cells induced glioma tumor growth by inhibiting tumor suppressor genes via miR-21 modulation^56^. These studies utilized a simple *in vitro* system to assay the interactions of microglia and tumor cells and verify the underlying mechanisms in animal tumor models. The present study used exosomes isolated from BV2 and RAW264.7 cells to assess the distinct roles of resident microglia versus infiltrating macrophages in brain tumor progression and migration. The results demonstrate the unique impact of microglia on tumor proliferation and migration. Further study should be conducted in orthotopic brain tumor models to verify the mechanism of microglia-mediating tumor progression and invasion, such as miR-24-3p-related signaling.

In summary, this study revealed the heterogeneous spatial distribution of infiltrating macrophages and resident microglia in brain TME. Further, it also discovered the distinct role of resident microglia over infiltrating macrophages on brain tumor progression. Microglia secreted sEVs and promoted the proliferation of brain tumor cells. Further, microglia-derived sEVs caused cell morphological change, thus enhancing cell migration. Microlia-mediated cell proliferation and mobility might be associated with VEGF and DEDD signaling pathways. This study demonstrates the vital function of microglia and reveals the mechanism for microglia-mediated tumor progression, illustrating a potential target for brain cancer therapy.

## Figures and Tables

**Table 1:** Primer sequences for RT-PCR.

Gene	Sequence
GAPDH-Forward	GGGCATCTTGGGCTACACT
GAPDH-Reverse	GGCATCGAAGGTGGAAGAGT
VEFG-A-Forward	ACTTGTGTTGGGGGGAGGATGTC
VEFG-A-Reverse	AATGGGTTTGTCGTGTTTCTGG
DEDD-Forward	AGCGGGAACTGCTGTCGTA
DEDD-Reverse	GGCCCAGTCCTCTGTAAGTTTG

## Abbreviations

TME: tumor microenvironment
ECM: extracellular matrix
EVs: extracellular vesicles
TAMs: tumor-associated macrophages
VEGF-A: vascular endothelial growth factor-A
DEDD: death effector domain-containing protein
